# A DCS-related lncRNA signature predicts the prognosis and chemotherapeutic response of patients with gastric cancer

**DOI:** 10.1042/BSR20220989

**Published:** 2022-09-02

**Authors:** Yang Zhang, Leyan Li, Yi Tu, Zongfeng Feng, Zhengrong Li, Yi Cao, Yong Li

**Affiliations:** 1 Department of General Surgery, The First Affiliated Hospital of Nanchang University, Nanchang, China; 2 Laboratory of Digestive Surgery, The First Affiliated Hospital of Nanchang University, Nanchang, China; 3 Queen Mary School, Medical Department of Nanchang University, Nanchang, China; 4 Department of Pathology, The First Affiliated Hospital of Nanchang University, Nanchang, China

**Keywords:** chemotherapy, DCS, gastric cancer, immune infiltration, lncRNA, prognosis signature

## Abstract

The combination of docetaxel, cisplatin, and S-1 (DCS) is a common chemotherapy regimen for patients with gastric cancer (GC). However, studies on long noncoding RNAs (lncRNAs) associated with the chemotherapeutic response to and prognosis after DCS remain lacking. The aim of the present study was to identify DCS mRNAs-lncRNAs associated with chemotherapy response and prognosis in GC patients. In the present study, we identified 548 lncRNAs associated with these 16 mRNAs in the TCGA and GSE31811 datasets. Eleven lncRNAs were used to construct a prognostic signature by least absolute shrinkage and selection operator (LASSO) regression. A model including the 11 lncRNAs (LINC02532, AC007277.1, AC005324.4, AL512506.1, AC068790.7, AC022509.2, AC113139.1, LINC00106, AC005165.1, MIR100HG, and UBE2R2-AS1) associated with the prognosis of GC was constructed. The signature was validated in the TCGA database, model comparison, and qRT-PCR experiments. The results showed that the risk signature was a more effective prognostic factor for GC patients. Furthermore, the results showed that this model can well predicting chemotherapy drug response and immune infiltration of GC patients. In addition, our experimental results indicated that lower expression levels of LINC00106 and UBE2R2-AS1 predicted worse drug resistance in AGS/DDP cells. The experimental results agreed with the predictions. Furthermore, knockdown of LINC00106 or UBE2R2-AS1 can significantly enhanced the proliferation and migration of GC AGS cells *in vitro*. In conclusion, a novel DCS therapy-related lncRNA signature may become a new strategy to predict chemotherapy response and prognosis in GC patients. LINC00106 and UBE2R2-AS1 may exhibit a tumor suppressive function in GC.

## Introduction

Gastric cancer (GC) remains the third most common cause of death among malignancies worldwide [1]. Although many treatments are available for GC, the prognosis after treatment is not optimistic, particularly for patients with advanced GC [2,3]. Many studies have confirmed that chemotherapy resistance is one of the reasons for unsatisfactory treatment results [4,5]. Accordingly, exploring the new molecular mechanism of chemotherapy resistance targets is urgently needed to improve outcome.

The number of studies focusing on long noncoding RNAs (lncRNAs) is increasing continuously [6]. One study found that the lncRNA maternally expressed gene 3 (MEG3) can inhibit GC cell proliferation and metastasis [7]. In addition, the lncRNA cancer susceptibility candidate 11 (CASC11) was found to not only inhibit GC cell apoptosis *in vitro* [8] but also promote growth and metastasis in colorectal cancer [9]. Furthermore, the lncRNA plasmacytoma variant 1 (PVT1) reportedly to promote gastric tumour growth *in vivo* by activating the STAT3/VEGFA axis [10]. These studies indicate that lncRNAs play pivotal roles in GC tumorigenesis.

More importantly, some lncRNAs are not only involved in GC development but also related to the resistance to various chemotherapy drugs [11,12]. Recent studies have that lncRNA colorectal neoplasia differentially expressed (CRNDE) could combat the growing threat of chemotherapy resistance in GC by regulating autophagy [13]. The lncRNA urothelial cancer associated 1 (UCA1) enhances cisplatin resistance via the miR-513a-3p/CYP1B1 axis [14]. The lncRNA forkhead box D1 antisense RNA 1 (FOXD1-AS1) exacerbates GC cell chemoresistance via PIK3CA/PI3K/AKT/mTOR signaling [15]. Therefore, lncRNAs play crucial roles in the chemotherapy resistance of GC. However, integrated studies on lncRNAs in GC-associated chemoresistance remain limited. In particular, no studies have investigated the targeting docetaxel, cisplatin, and S-1 (DCS) therapy-related lncRNAs.

The main objective of the present study was to explore the lncRNAs associated with DCS treatment using the Gene Expression Omnibus (GEO) and The Cancer Genome Atlas (TCGA) databases. Moreover, a prognostic model composed of lncRNAs was constructed to predict long-term outcomes in patients with GC, and an assessment scheme for individual chemotherapy therapeutic strategies was established.

## Methods

### Cell culture

The human gastric adenocarcinoma cell lines AGS and AGS/DDP were purchased from KeyGEN Biotechnology Company (Nanjing, Jiangsu, China). The cells were cultured in RPMI-1640 medium containing 10% FBS and placed in an incubator with a constant temperature of 37°C and 5% carbon dioxide. AGS/DDP cells were cultured in complete medium containing 500 ng/mL DDP (Sigma, St. Louis, MO, U.S.A.) in RPMI-1640 to maintain cell drug resistance.

### Quantitative real-time polymerase chain reaction (qRT-PCR)

TRIzol (Invitrogen; Thermo Fisher Scientific, Inc., Waltham, M, U.S.A.) was used for the extraction of total RNA from cells. cDNA was synthesized using cDNA Synthesis Kit (TransGen Biotech, Inc., Beijing, China) according to the manufacturer’s instruction. qRT-PCR was performed using the PowerUp SYBR Green Master Mix (TransGen Biotech, Inc., Beijing, China) according to the manufacturer’s instruction. The data were collected using an ABI Prism 7500 Sequence Detection system (Applied Biosystems; Thermo Fisher Scientific, Inc). GAPDH was used as normalization control for relative quantification in the qRT-PCR analysis. Relative quantitative analysis was performed with the 2^−ΔΔCt^ method. The primer sequences were amplified using the following primers: Human LINC00106 (F, GGTCACCTGAGATGGAGCAG; and R, CGTCTGTCTTACGGCACGAA), Human UBE2R2-AS1 (F, ACTCGTTCCACCCTTTGTGG; and R, TAGGACGCTGCAGTGAATCC), and Human GAPDH (F, GGAGCGAGATCCCTCCAAAAT; and R, GGCTGTTGTCATACTTCTCATGG).

### siRNAknockdown

Cells were seeded in six-well plates at a density of 3.0 × 10^5^/well. Lipofectamine 2000 Reagent (Invitrogen, Carlsbad, CA, U.S.A.) was used for the transfection of predesigned human LINC00106/UBE2R2-AS1 siRNAs and the siRNA negative control (GenePharma, Suzhou, China) into AGS cells. The primer sequences used were as follows: si-LINC00106 sequence, sense 5′-GGGAAGACUUCAGGCUUCATT-3′ and antisense 5′-UGAAGCCUGAAGUCUUCCCTT-3′; and si-UBE2R2-AS1 sequence, sense 5′-GGAAGCUAUCAGUCUCCCUTT-3′ and antisense 5′-AGGGAGACUGAUAGCUUCCTT-3′. The nontargeting control siRNA was used as a negative control (si-NC). All steps were performed following the manufacturer’s protocol.

### Cell proliferation assays

Cell proliferation was determined using the Cell Counting Kit-8 (Sigma, St. Louis, MO, U.S.A.) based on the manufacturer’s instructions. During CCK-8 detection, 1 × 10^4^ cells/well were inoculated in 96-well plates. After 72 h, 10 μl of Cell Counting Kit solution was added to each well, and the plates were incubated at 37°C for 4 h. The absorbance values at 450 nm were then measured using a microplate reader (Thermo, U.S.A.). The experiments were repeated at least three times.

### Transwell migration assay

The treated AGS cells (2.0 × 10^5^/ml) were added to the upper chambers of a Transwell (BD Biosciences, NY, U.S.A.). FBS (10%) was added to the lower chambers. After incubation for 48 h, the nonmigrated cells were removed, and the migrated cells were stained with 0.1% Crystal Violet solution. The sections were visualized under an inverted fluorescence microscope (magnification at ×100).

### Preparation of data

Transcriptome and clinical data were obtained from The Cancer Genome Atlas (TCGA) (https://tcga-data.nci.nih.gov/tcga/) database, and the expression microarray dataset GSE31811 was obtained from The Gene Expression Omnibus (GEO) (https://www.ncbi.nlm.nih.gov/geo) database.

### Identification of DCS therapy-associated mRNAs-lncRNAs

Ninety up-regulated DCS-associated mRNAs were identified from GSE31811 (*P* value <0.01 and fold-change > 2.0). Sixteen differentially expressed mRNAs and 548 lncRNAs related to these mRNAs were obtained from TCGA differential expression data (*P* value <0.001 and calculated Pearson correlation coefficient > 0.4).

### Establishment of the DCS therapy-associated lncRNAs prognostic risk model

**Figure 1:**
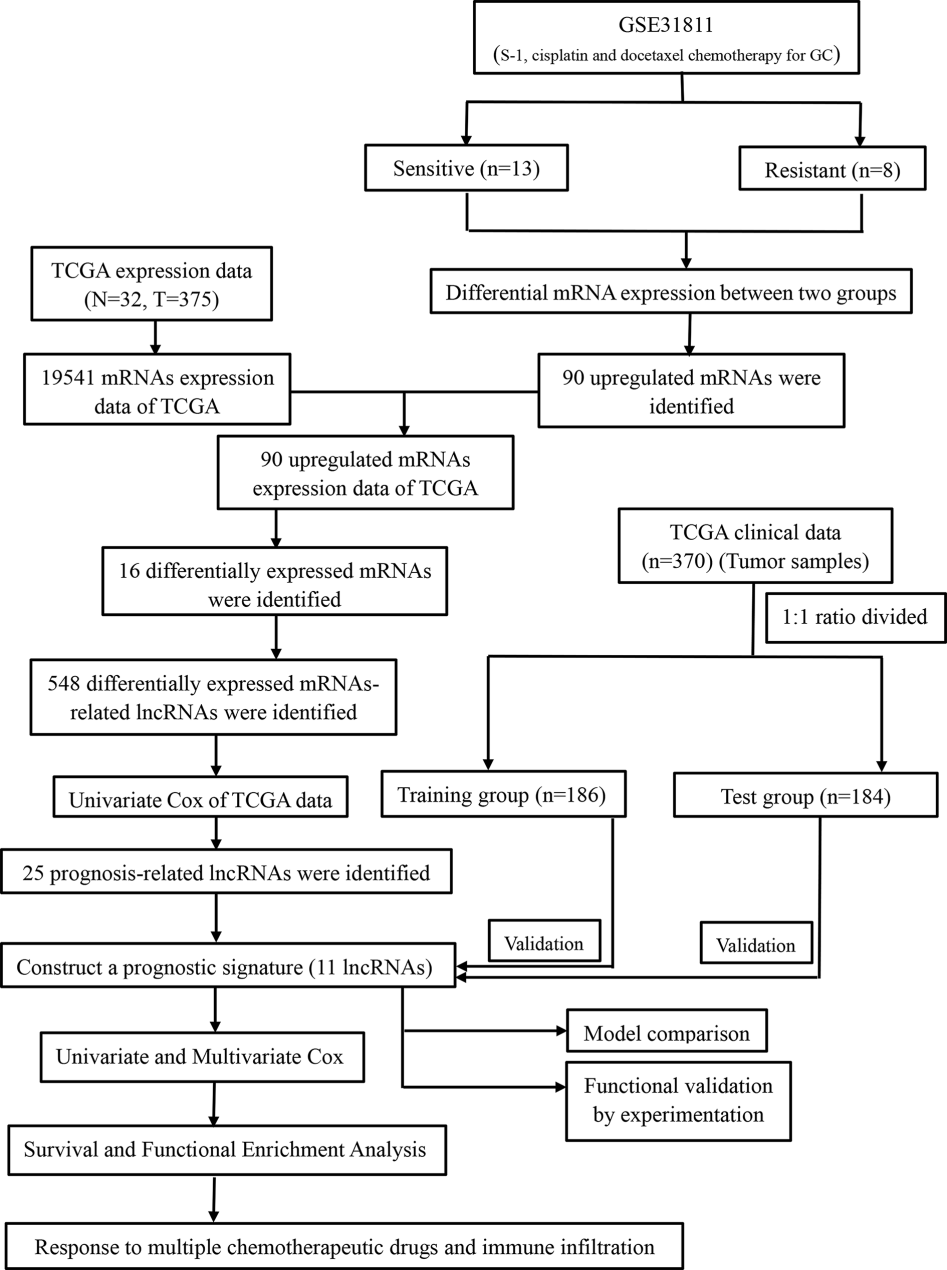
Graphical flowchart of this work

**Figure 2:**
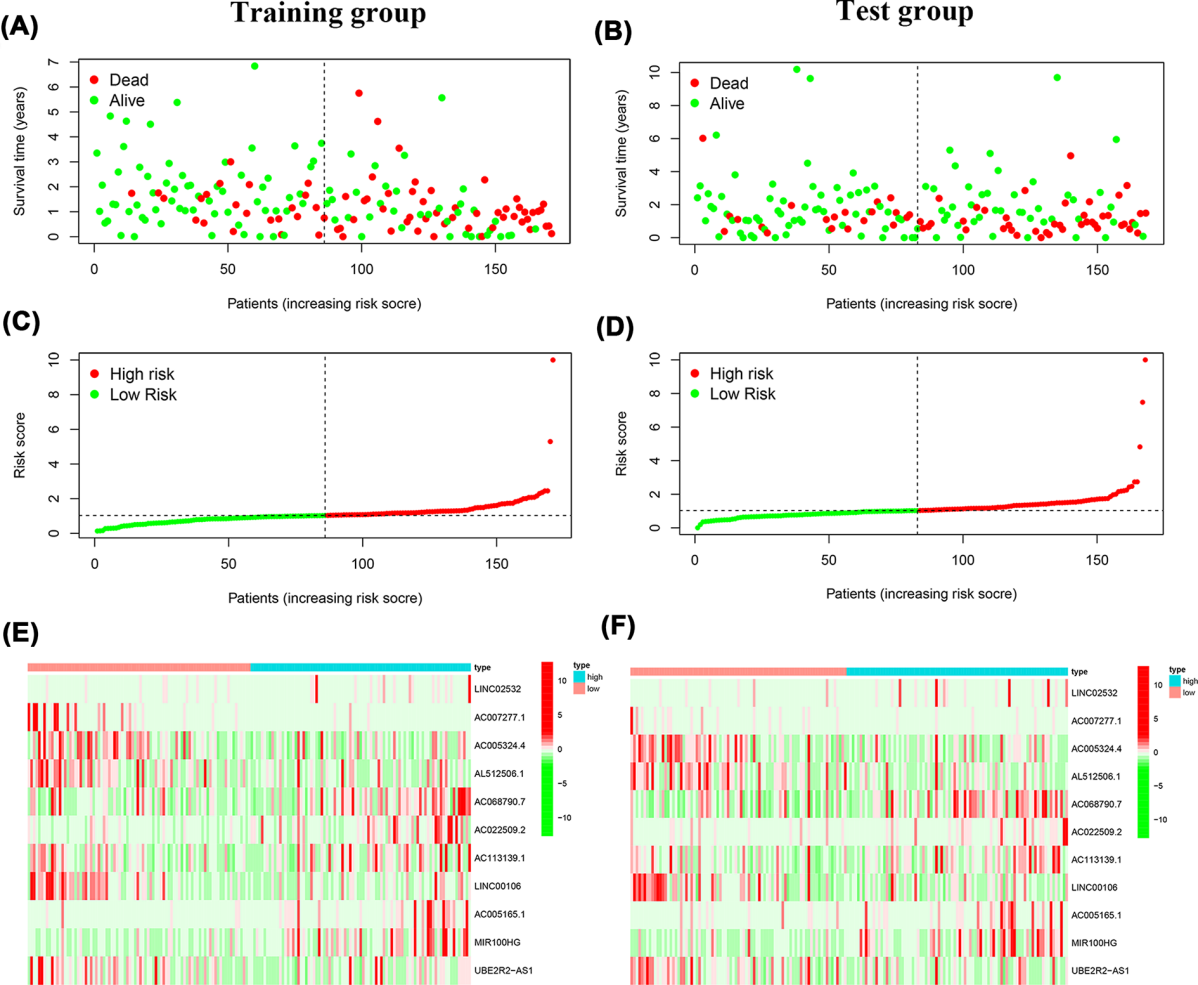
The risk scores analysis of the prognostic signature model in the training group and test group (**A**) The distributions of risk score and OS status in the training group. (**B**) The distributions of risk score and OS status in the test group. (**C**) The distribution of the risk scores in the training group. (**D**) The distribution of the risk scores in the test group. (**E**) Prognostic signature signal heatmaps in the training group. (**F**) Prognostic signature signal heatmaps in the test group.

A total of 370 patients with GC from TCGA were randomly divided at a 1:1 ratio into a training group and test group to validate the DCS-related lncRNA signature. Univariate Cox regression analysis was performed to screen the prognostic lncRNAs in the training group. A total of 25 prognostic lncRNAs were identified (Figure 2). The selected differentially expressed prognostic lncRNAs were then identified as candidate lncRNAs for the model. A model incorporating 11 lncRNAs with optimal GC correlation and prognosis was constructed based on LASSO Cox regression. The prognostic signature risk score was as follows: Risk Score = e ^sum (expression of each lncRNA × corresponding regression coefficient)^. The details are shown in Figure 1.

### Analysis of the clinicopathological characteristics associated with survival

The significance of clinicopathological features and overall survival (OS) in the training and test groups was analyzed by univariate and multivariate Cox regression analyses. A differential survival analysis of clinically relevant characteristics in the high- and low-risk groups was performed. We performed receiver operating characteristic (ROC) analysis and calculated the area under the curve (AUC) to assess the accuracy of the model. The consistency index (C-index) was used to compare the predictive performance of the different models.

### Nomogram

Nomograms were used to calculate the total scores and predict the 1-, 3-, and 5-year survival probabilities. Calibration curves and DCA were used to compare net benefits with different predictions. Details of the methodology were previously described [16–18]

### Functional enrichment analysis

The annotated c2.cp.kegg.v7.2.symbols.gmt gene set in the ‘Molecular Signature Database’ in GSEA version 3.0 (http://software.broadinstitute.org/gsea/downloads.jsp) was selected [19]. Whole-transcriptome data from all tumor samples were used for GSEA. The critical criteria are as follows: normalized enrichment score >1.0 and *P*<0.05.

### Chemosensitivity prediction

We used the ‘pRRophetic’ software package in R software (4.1.0) to obtain IC50 values for common chemotherapeutic and targeted drugs. The methods and strategies are derived from previous studies [20–22]. The Mann–Whitney *U* test was used to compare the IC50 values between the two groups.

### Different models and immune infiltration analysis

We used the restricted mean survival (RMS) package [23] to calculate the Concordance index (C-index) of different prognostic signatures. In addition, the ssGSEA method of the R software Gene Set Variation Analysis (GSVA) package [24] was used to analyze the infiltration level of different immune cells and immune functions in the high- and low-risk groups.

### Common transcription factor prediction

The 11 lncRNAs and promoter regions of Homo sapiens were analyzed with the NCBI gene browser (https://www.ncbi.nlm.nih.gov/gene/) [25]. The potential transcription factors that bound to the promoter region were then predicted using PROMO 3.0 (http://alggen.lsi.upc.es/cgi-bin/promo_v3/promo/promoinit.cgi?dirDB=TF_8.3) [26].

### Statistical analysis

R statistical software 4.1.0 and GraphPad Prism 8.0 were used for all statistical analyses. The Mann–Whitney *U* test and Spearman correlation were used to analyze the correlations between the high- and low-risk groups. *P* values for the differences between two groups were calculated by Student’s *t*-test (two tailed). A *P* value less than 0.05 was considered to indicate statistical significance.

## Results

### Identification of prognostic DCS therapy-associated mRNAs-lncRNAs in GC

Of the patients in the GSE31811 dataset, 13 and 8 patients were identified as DCS-sensitive and DCS-resistant patients, respectively. Sixteen differentially expressed mRNAs and 548 differentially expressed mRNAs-lncRNAs were obtained from GEO data and TCGA data (Figure 1). Further univariate Cox analysis was performed, and 25 lncRNAs associated with DCS were preliminarily screened and found to be significantly related to the OS of patients with GC (Supplementary Figure S1a). The details are shown in Supplementary Table S1 and Supplementary File S1.

### Construction a DCS therapy-associated lncRNA prognostic signature

A total of 370 patients with GC from TCGA were enrolled in 1 of 2 cohorts (training group [*n*=186] and test group [*n*=184]). Eleven lncRNAs were identified and used for model construction: LINC02532, AC007277.1, AC005324.4, AL512506.1, AC068790.7, AC022509.2, AC113139.1, LINC00106, AC005165.1, MIR100HG, and UBE2R2-AS1 (Supplementary Figure S1c).

### Relationship between prognostic model risk score and clinicopathological features

**Figure 3:**
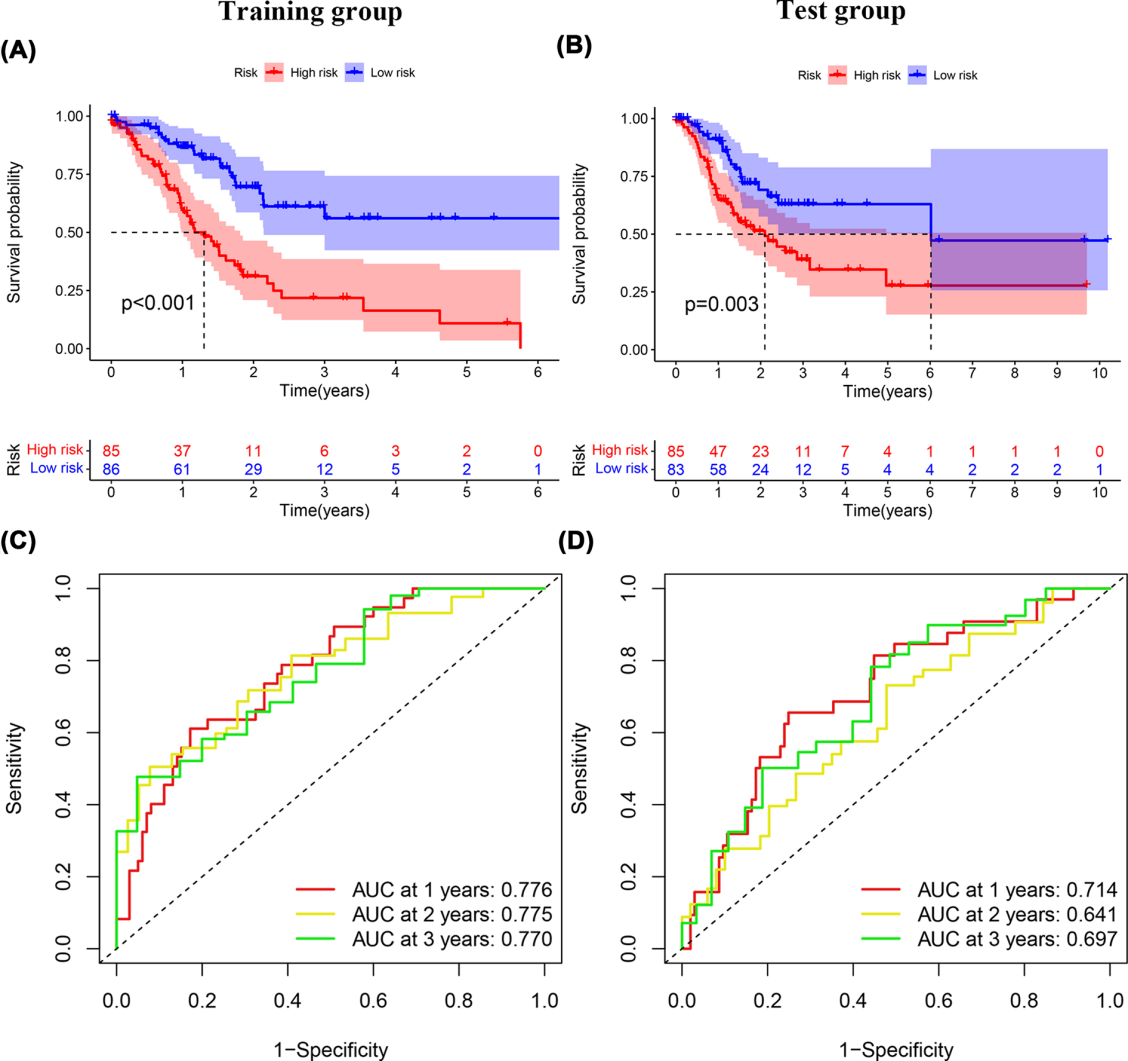
K-M survival and ROC curve analysis (**A**) Survival curve of the patients classified in high- and low-risk training group. (**B**) Survival curve of the patients classified in high- and low-risk test group. (**C**) AUC of time-dependent ROC curves verified the prognostic accuracy of risk scores in the training group. (**D**) AUC of time-dependent ROC curve verified the prognostic accuracy of the risk score in the test group.

A risk score dot plot was constructed, and the results for both the training and test groups indicated that the high-risk group was characterized by more deaths (Figure 2A–D). The expression of 11 DCS therapy-related lncRNAs (Figure 2E,F) is shown in the heatmap. A Kaplan–Meier (K-M) survival curve analysis was conducted to assess OS in GC patients. We found significantly lower OS rates in the high-risk group (*P*<0.001 and *P*<0.003; Figure 3A,B). We also performed ROC curve analysis to assess the prognostic model at 1, 2, and 3 years. The AUC values of the training group were 0.776 for 1 year, 0.775 for 2 years, and 0.770 for 3 years, and those of the test group were 0.714 for 1 year, 0.641 for 2 years, and 0.697 for 3 years (Figure 3C,D).

### Clinical prognostic value of the DCS therapy-associated lncRNA signature

**Figure 4:**
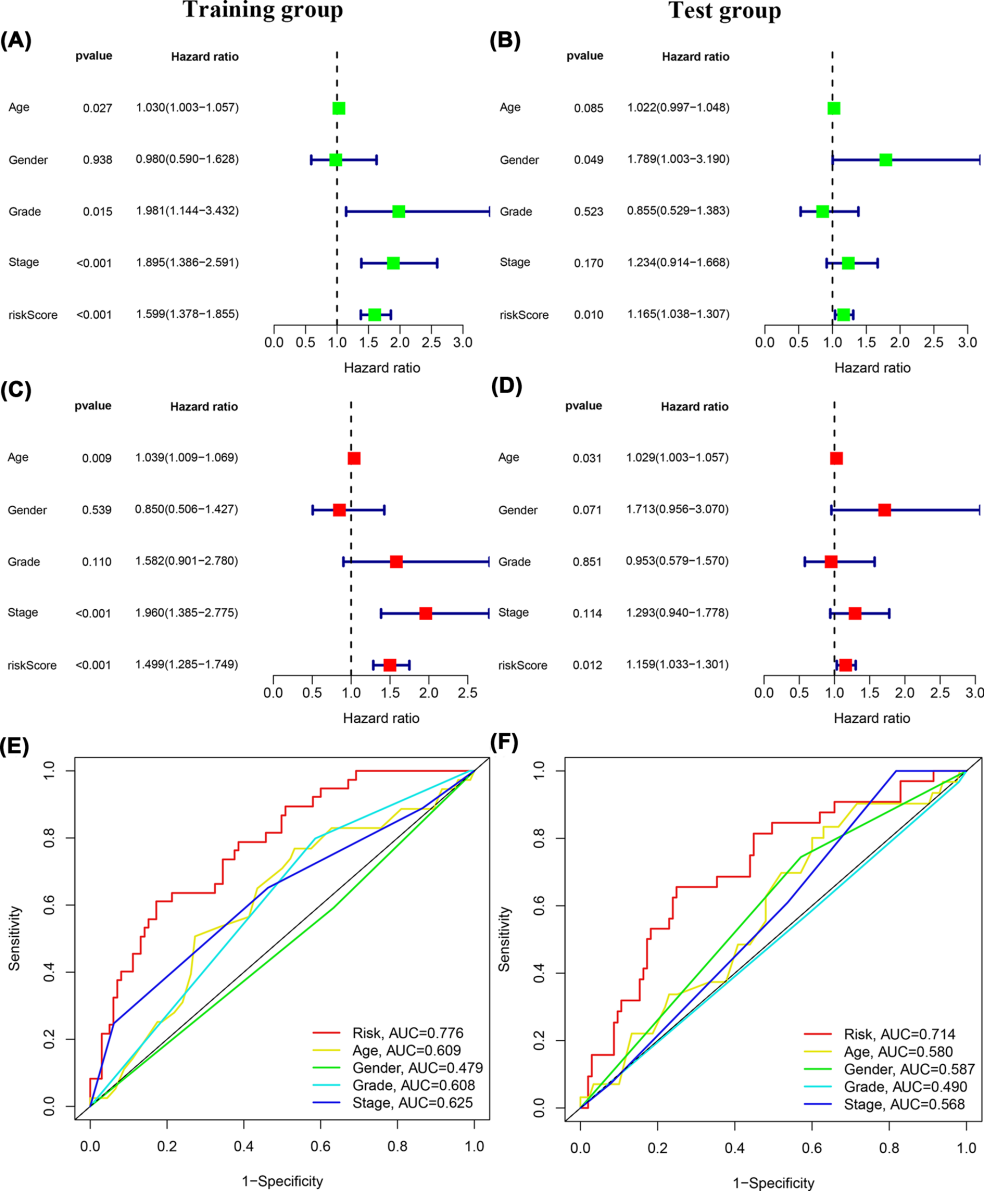
K-M survival and ROC curve analysis (**A**) Univariate cox regression analysis results of OS in training group. (**B**) Univariate cox regression analysis results of OS in test group. (**C**) Multivariate cox regression analysis results of OS in training group. (**D**) Multivariate cox regression analysis results of OS in test group. (**E**) The AUC of ROC curve was used to compare the prognostic accuracy of risk scores and related clinical factors in the training group. (**F**) The AUC of ROC curve was used to compare the prognostic accuracy of risk scores and related clinical factors in the test group.

**Figure 5:**
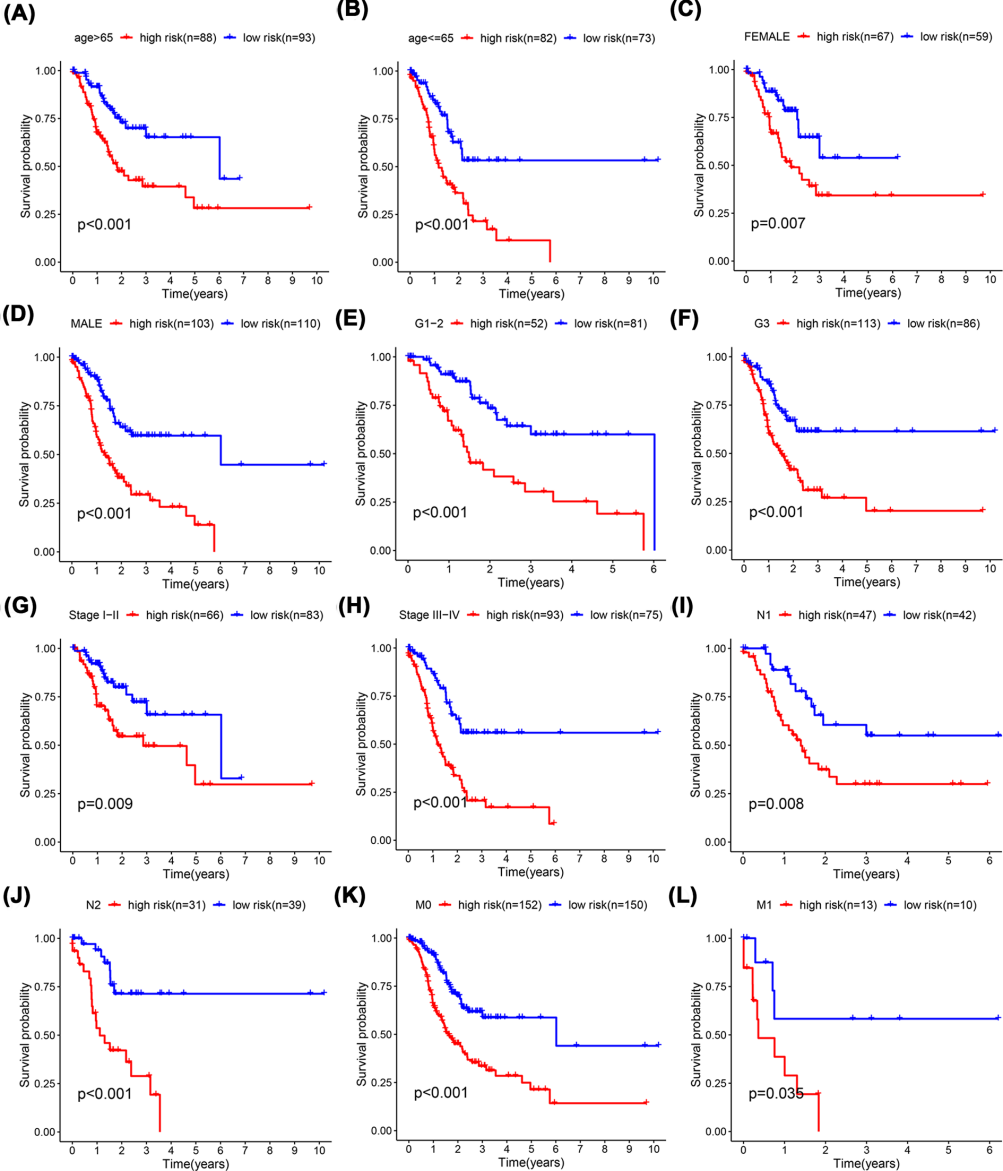
K-M survival curves were analyzed for clinical prognostic factors (**A**) Age >65 years and (**B**) ≤65 years. (**C**) Women and (**D**) men. (**E**) G1+G2 and (**F**) G3. (**G**) Stage I+II and (**H**) Stage III+IV. (**I**) N1 and (**J**) N2. (**K**) M0 and (**L**) M1

The Univariate Cox regression analysis indicated that the risk score was significantly associated with OS in both the training group (HR = 1.599, 95% CI: 1.378–1.855, *P*<0.001) and the test group (HR = 1.165, 95% CI: 1.038–1.307, *P*=0.008) (Figure 4A,C). The multivariate Cox regression analysis results revealed that the signature risk score remained a significant independent predictor of OS (training group: HR = 1.499, 95% CI: 1.265–1.749, *P*<0.001; test group: HR 1.159, 95% CI: 1.033–1.301, *P*=0.012; Figure 4B,D). The ROC curve analysis indicated that the DCS-related lncRNA signature prediction attained AUC values of 0.776 (training group) and 0.714 (test group). The AUC value of the signature was greater than that of other clinical prognostic factors (Figure 4E,F). Furthermore, a K-M survival analysis was performed for each subgroup isolated based on clinicopathological features, including age, sex, N stage, M stage, and clinical grade and stage (Figure 5A–L). The results showed that the high-risk group had a significantly worse OS than the low-risk group, as revealed after stratification by age, sex, N stage, M stage, and clinical grade and stage.

### Prognostic nomogram

**Figure 6:**
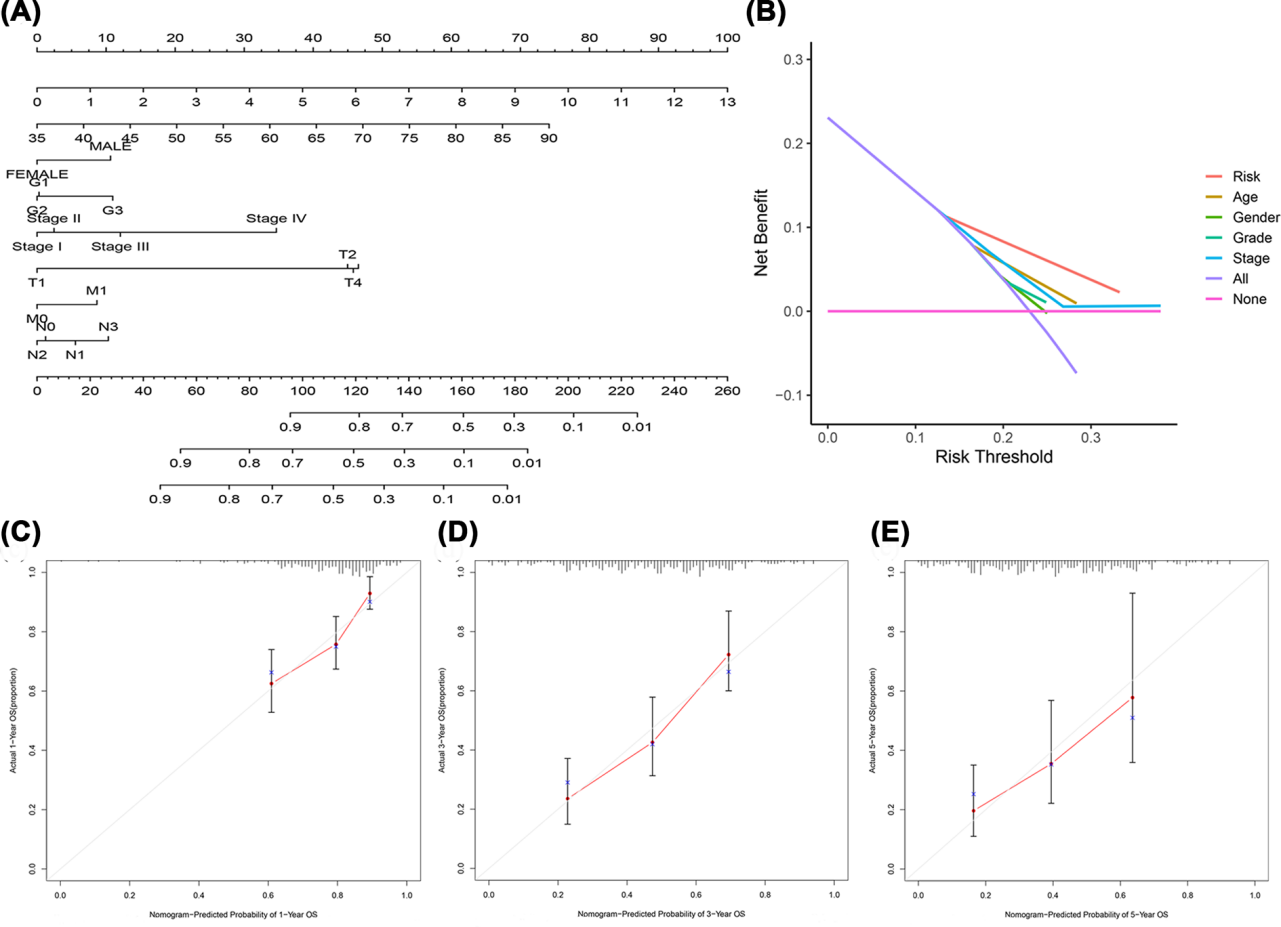
Prognostic nomogram with patients with GC (**A**) Nomogram of the prognostic model. (**B**) Decision curve analysis of the 1- (**C**), 3- (**D**), and 5-year. (**E**) The accuracy of assessing the prediction nomogram.

In the present study, a nomogram based on clinicopathological features and the 11 DCS treatment-related lncRNA prognostic signature was used to evaluate the clinical utility of the model (Figure 6A). Higher risk scores denote worse performance. The DCA results (Figure 6B) indicated that these nomogram predictions agreed well with clinical applicability for predicting the prognosis of patients with GC treated with DCS. Finally, calibration curves were constructed to evaluate the agreement between the actual observed OS rate and the OS rate predicted by the nomogram. The results showed that the predicted probability of OS at 1, 3, and 5 years was relatively good (Figure 6C–E).

### Kyoto Encyclopedia of Genes and Genomes enrichment analysis

To further analyze the differences in biological functions between the high- and low-risk groups, a Kyoto Encyclopedia of Genes and Genomes pathway enrichment analysis was conducted. The top 10 enrichment pathways in the high- and low-risk groups are listed in Supplementary Figure S2, and detailed parameters are provided in Supplementary Table S3.

### Responses to chemotherapy and immune infiltration

**Figure 7:**
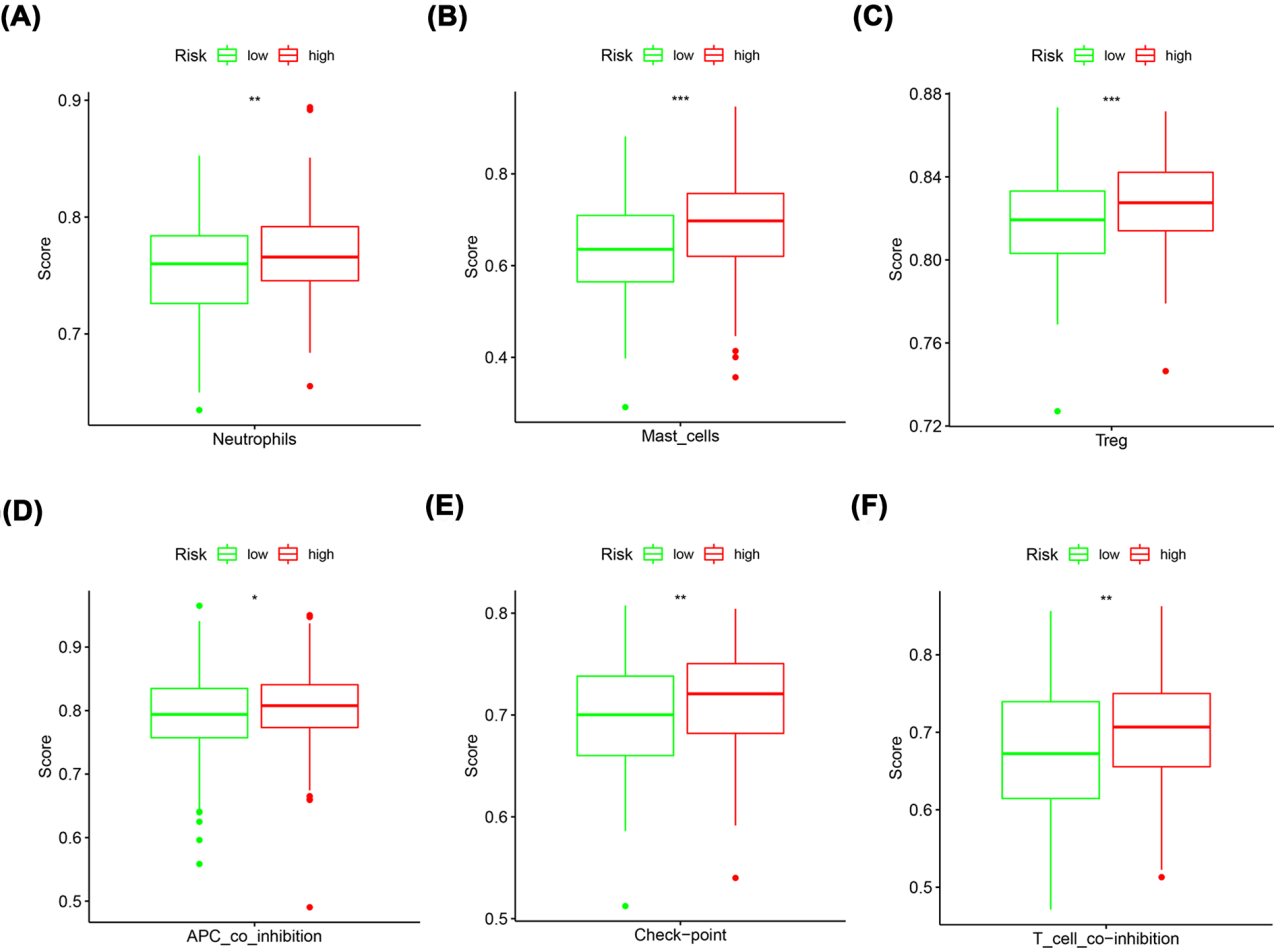
Using the ssGSEA method to analyze the infiltration level of different immune cells and function between high- and low-risk group (**A–C**) Boxplot showed the different proportions of immune-infiltrating cells between high- and low-risk group. (**D–F**) Boxplot showed the different proportions of immune-infiltrating function between high- and low-risk group; *P<0.05; **P<0.01; ***P<0.001.

The pRRophetic algorithm was subsequently used to predict the IC50 values of the different chemotherapeutic agents tested in the present study, including cisplatin, paclitaxel, vinblastine, dimethyloxalylglycine (DMOG), 5-aminoimidazole-4-carboxamide riboside (AICAR), all-trans retinoic acid (ATRA), axitinib, pazopanib, and imatinib (Supplementary Figure S3). Significant differences in sensitivity to nine chemotherapy drugs were found between the high- and low-risk groups (Supplementary Figure S3c and S3i). More interestingly, the analysis of immune infiltration results found that neutrophils, mast cells, and Treg cells had significantly higher immune cell scores in the high-risk group than in the low-risk group (Figure 7A–C). In addition, the immune function scores of APC coinhibition, Check point, and T-cell coinhibition obtained for the high-risk group were significantly higher than those found for the low-risk group (Figure 7D–F).

### Comparison of five prognostic risk models

**Figure 8:**
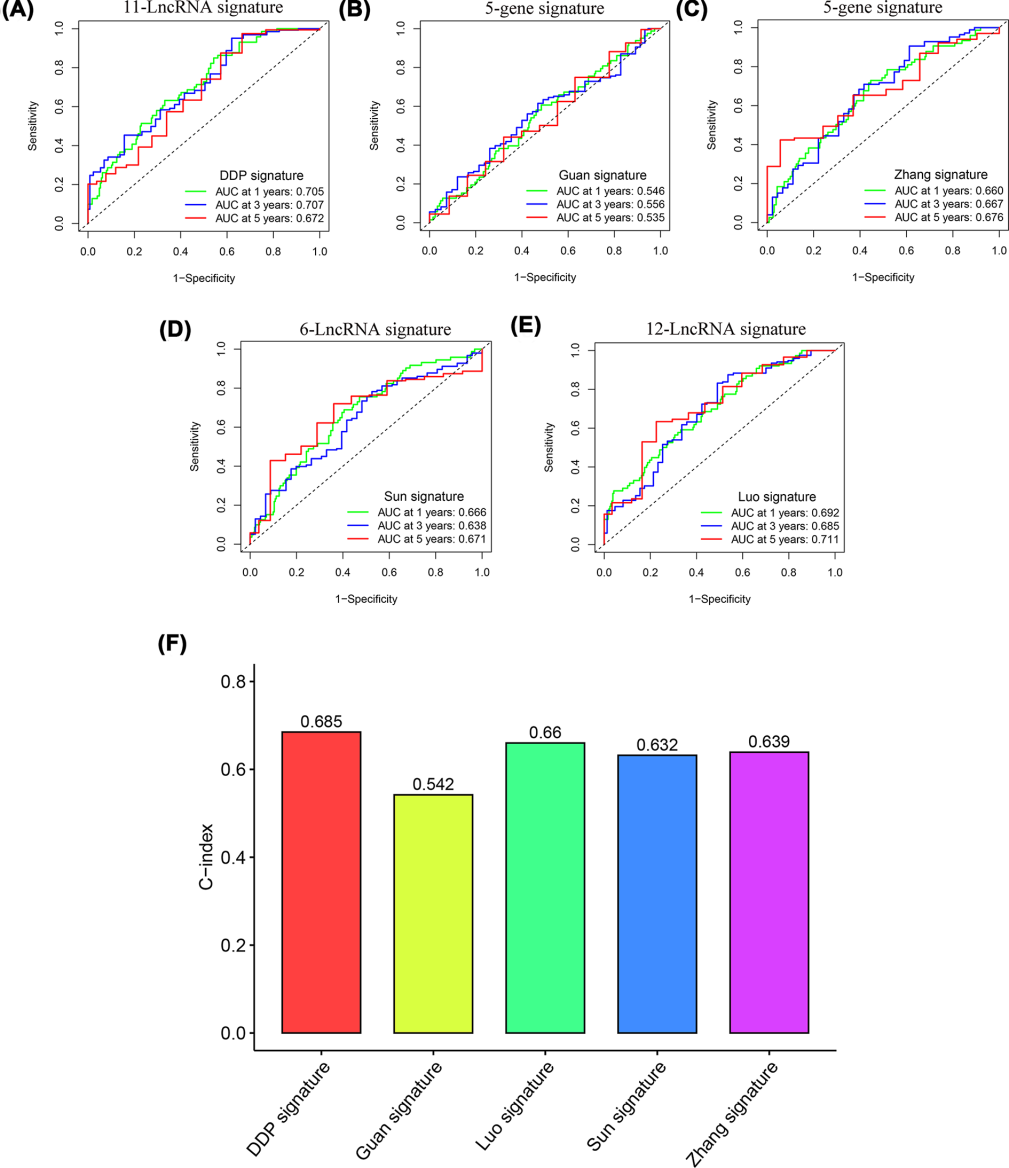
Comparison of prognostic risk models (**A–E**) ROC curves of our DDP signature and four other published signatures. (**F**) Comparison of Concordance index (C-index) of the five prognostic risk models.

To compare the predictive performance of our 11 lncRNA markers with other models, we selected four other reported risk models: 5 gene markers [27], 5 gene markers [28], 12 LncRNA markers [29], and 6 LncRNA markers [30]. Notably, a comparison of the predicted values of the AUC models from 1 to 3 years revealed that our DDP model had the highest predicted value (Figure 8A–E). In addition, our model had the highest C-index with 0.685 (Figure 8F).

### Common transcription factors

We predicted a total of 20 common transcription factors on the promoters of the 11 lncRNAs by bioinformatics methods. Among these common transcription factors, the transcription factor interferon regulatory factor-1 (IRF-1) can reverse chemoresistance by down-regulating the expression of P-glycoprotein in GC [31]. Abacavir induces the transcriptional activity of the transcription factor Yin Yang 1 (YY1) in GC cells [32]. Furthermore, drugs capable of inhibiting YY1-mediated transcription have been identified as suitable targeted therapeutic candidates for gastric tumors [33]. The expression of miR-100 induced by the transcription factor CCAAT/enhancer-binding protein beta (C/EBPα) suppresses tumor metastasis and growth by targeting ZBTB7A in GC [34]. In addition, C/EBPβ regulates homeostatic and oncogenic gastric cell proliferation [35]. These findings confirm that these transcription factors play an important role in the progression of GC. These transcription factors are provided in Supplementary File S2.

### Validation of the expression of LINC00106 and UBE2R2-AS1 in AGS and AGS/DDP cells

**Figure 9:**
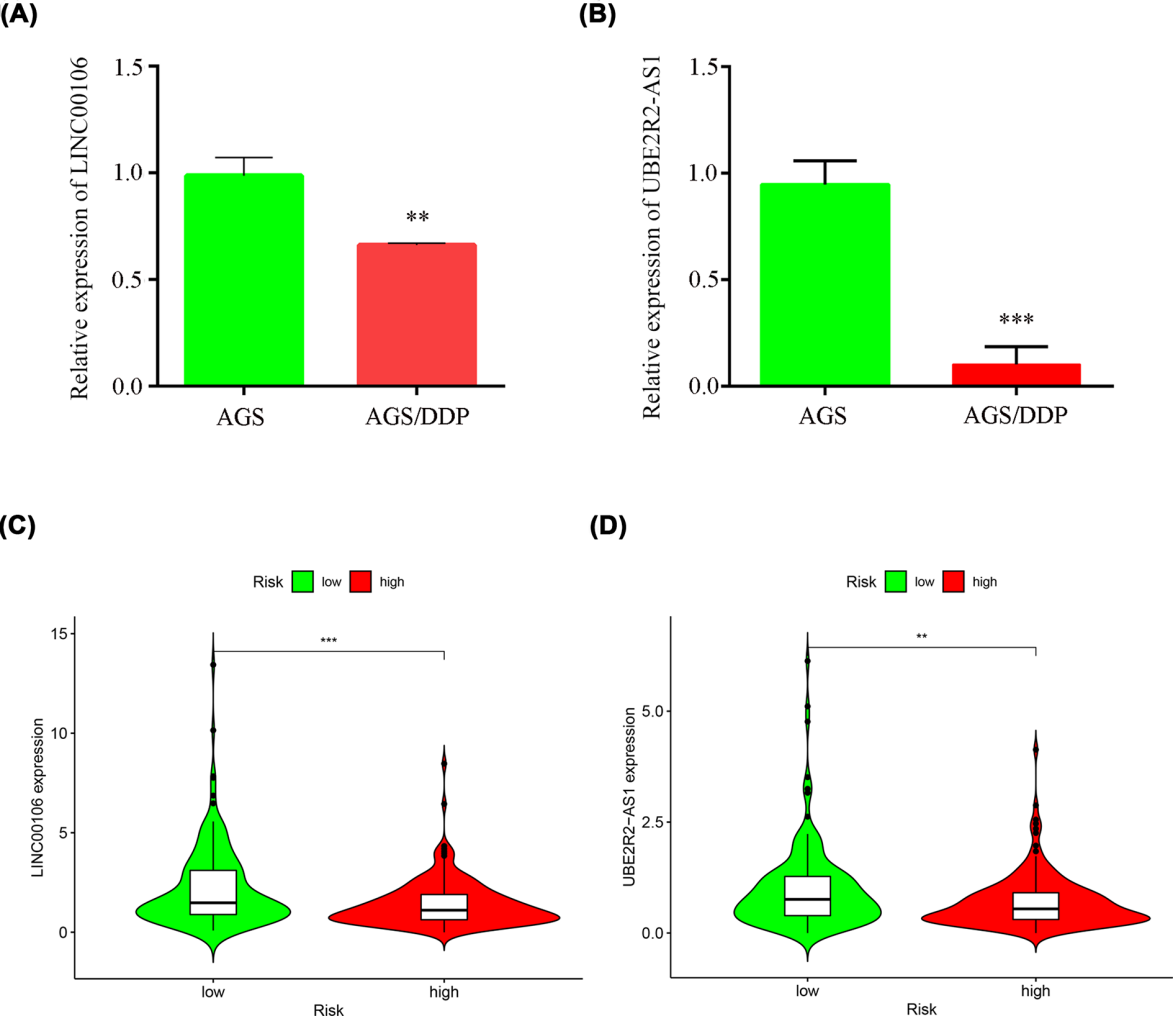
Validation the expression of lncRNAs (**A**) LINC00106, (**B**) UBE2R2-AS1 in AGS and AGS/DDP cells. (**C**) LINC00106, (**D**) UBE2R2-AS1 in high-risk group and low-risk group. **P<0.01; ***P<0.001.

We assess the expression levels of the LINC00106 and UBE2R2-AS1 lncRNAs in GC cell lines by qRT-PCR. As shown in ( Figure 9A,B), the expression of LINC00106 and UBE2R2-AS1 was decreased in AGS/DDP cells. Their expression levels were also lower in the high-risk group (Figure 9C,D). The expression results were consistent with the bioinformatics analysis (Supplementary Figure S3a).

### Effects of LINC00106 and UBE2R2-AS1 on GC cell proliferation and migration

**Figure 10:**
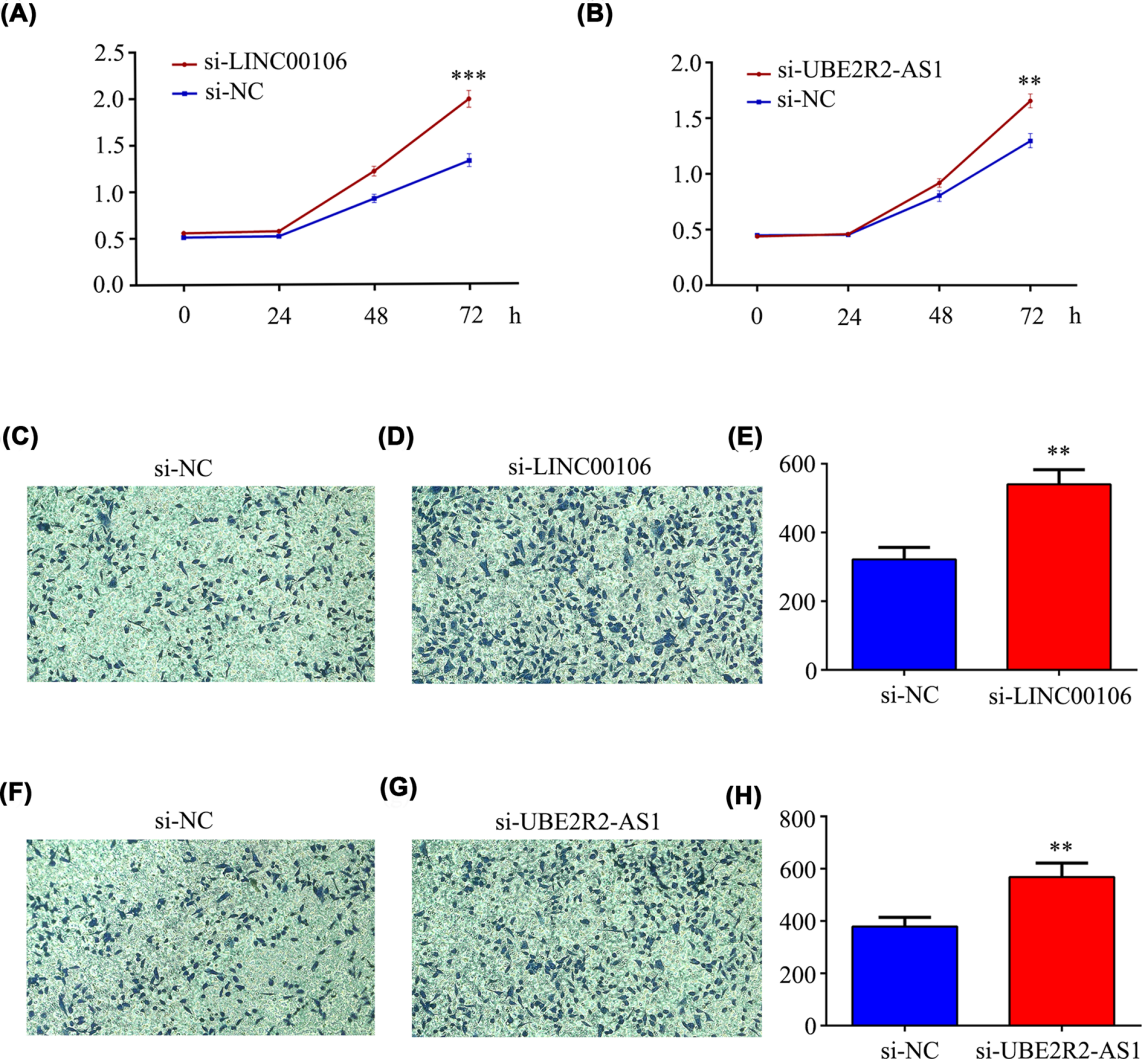
The effects of LINC00106 and UBE2R2-AS1 on GC AGS cells proliferation and migration (**A,B**) Effect of si-LINC00106 and si-UBE2R2-AS1 transfection on proliferation of AGS cells. (**C–H**) Effect of si-LINC00106 and si-UBE2R2-AS1 transfection on migration of AGS cells.

To understand the biological functions of LINC00106 and UBE2R2-AS1 in GC, we explored the effect of knocking down the expression of LINC00106 and UBE2R2-AS1 on the biological behavior of AGS GC cells. The CCK-8 experiment showed that the cell viability of AGS in the si-LINC00106 and si-UBE2R2-AS1 groups was significantly higher than that in the si-NC group (Figure 10A,B), and the migration ability of the cells in both groups was also significantly enhanced (Figure 10C,D). These results indicate that knockdown of LINC00106 or UBE2R2-AS1 can significantly enhance the proliferation and migration of AGS GC cells.

## Discussion

The DCS combination comprises chemotherapy drugs commonly used for patients with GC, and an increasing number of clinical studies have investigated these agents [36–38]. Thus, it is becoming more important to explore the mechanism of DCS chemotherapy resistance in patients with GC Therefore, identifying prognostic biomarkers for DCS chemotherapy resistance has become a focus of research.

Although many independent studies have investigated single lncRNAs in chemotherapeutic resistance [39–42], studies on the effect and function of lncRNAs in predicting chemotherapeutic resistance, particularly studies on prognostic models using DCS therapy-related lncRNAs, remain lacking. In the present study, the chip dataset GSE31811 for DCS-treated GC was mined using the GEO database. Data from samples of patients with chemotherapeutic drug treatment-resistant and treatment-susceptible GC were analyzed. A lncRNA risk score model predicting the chemotherapeutic resistance to DCS and prognosis in patients with GC was established.

A total of 548 lncRNAs related to these mRNAs were obtained from the TCGA expression database (Figure 1). Then, 25 differentially correlated lncRNAs were selected by univariate Cox analysis of the training group (Supplementary Figure S1). An 11 DCS therapy-related lncRNA prognostic model was constructed via LASSO regression analysis (Figure 2). The accuracy of the model was then confirmed with the training group and test group. The prognostic efficacy of the model for the OS status of patients with GC was evaluated (*P*<0.001 and *P*<0.003; Figure 3A,B). The AUC values of the training group were 0.776 for 1 year, 0.775 for 2 years, and 0.770 for 3 years, and the AUC values of the test group were 0.714 for 1 year, 0.641 for 2 years, and 0.697 for 3 years (Figure 3C,D). Furthermore, the AUC value of the signature was greater than that of other clinical prognostic factors (Figure 4E,F). To further evaluate the value of this prognostic model for DCS treatment in the clinical application of various clinicopathological features, a K-M survival analysis was performed for each subgroup isolated according to clinicopathological features. The results indicated that the OS rates of age, sex, N stage, M stage, clinical grades, and stages in the high-risk group showed poorer survival than those in the low-risk group (Figure 5). In the present study, the patients with higher risk scores based on the nomogram had worse performance (Figure 6). Using the nomogram, risk prediction could be more tailored to patients. The results herein showed that we built a risk score model based on an 11-lncRNA signature that had a powerful ability to predict the survival of patients with GC.

A KEGG enrichment analysis in the high- and low-risk groups showed that multiple signalling pathways, including hypertrophic cardiomyopathy, dilated cardiomyopathy, focal adhesion, extracellular matrix receptor interaction, and vascular smooth muscle contraction, might be involved. Several signaling pathways are closely associated with the occurrence and development of tumors [43–45]. However, the clear mechanism remains unclear and is one focus of our future efforts. Furthermore, we further analysed the correlation between prognostic characteristics and several major current chemotherapy agents. We calculated the IC50 values of nine chemotherapy drugs and targeted drugs (cisplatin, paclitaxel, vinblastine, DMOG, AICAR, ATRA, axitinib, pazopanib, and imatinib), and the results showed that the IC50 values of cytotoxic chemotherapy drugs and targeted drugs were higher in the low-risk group. These results further highlight the key role of this model in estimating the patient response to chemotherapy drugs (Supplementary Figure S3). In terms of immune infiltration, both immune cells and immune function related indexes had higher immune scores in the high-risk group than in the low-risk group (Figure 7). Interestingly, neutrophils [46], mast cells [47], and Treg cells [48] have been shown to promote the progression of GC. In addition, APC coinhibition [49], Check point [50], and T-cell coinhibition [51] are also important for the progression of GC. These results were in accordance with our finding. Taken together, our finding show that this model can well predict the chemotherapy drug response and immune infiltration in patients with GC.

In addition, we found that multiple studies have confirmed that the common transcription factors of these lncRNAs play an important role in the progression of GC. This finding further indicates that these lncRNAs may play a key role in GC. Among the 11 lncRNAs included in the model, UBE2R2-AS1 was confirmed to have an oncogenic function in hepatocellular carcinoma [52]. LINC00106 is indicated to promote stemness and metastasis in hepatocellular carcinoma cells [53]. In addition, we found lower expression levels of UBE2R2-AS1 and LINC00106 in AGS/DDP cell lines and high-risk groups (Figure 9). These results indicate that the group with lower LINC00106 and UBE2R2-AS1 expression levels exhibited worse drug resistance and worse prognosis. More importantly, our DDP model exhibited a higher C-index and predictive value than the other two gene models and two lncRNA models (Figure 8). Therefore, these results show further evidence of accuracy and advantages of our model. To further verify the molecular mechanisms involving LINC00106 and UBE2R2-AS1, we conducted functional experiments with AGS cells. The *in vitro* results showed that the down-regulation of LINC00106 and UBE2R2-AS1 markedly enhanced the proliferation and migration of AGS GC cells (Figure 10).

## Conclusion

Our studies have uncovered a new DCS therapy-related lncRNA signature that could accurately predict outcomes for patients with GC. More importantly, the knockdown of LINC00106 or UBE2R2-AS1 can significantly enhance the proliferation and migration of GC AGS cells.

## Supplementary Material

Supplementary Figures S1-S3 and Supplementary Tables S1-S3

Supplementary Files S1-S2

## Data Availability

All data used in the study can be downloaded from the TCGA (https://tcga-data.nci.nih.gov/tcga/) database and the GEO (https://www.ncbi.nlm.nih.gov/geo). The other datasets used and/or analyzed during the present study are available from the corresponding author on reasonable request.

## Abbreviations

AICAR: 5-aminoimidazole-4-carboxamide riboside
ATRA: all-trans retinoic acid
AUC: area under the curve
C/EBPα: CCAAT/enhancer-binding protein beta
DCS: docetaxel, cisplatin, and S-1
DMOG: dimethyloxalylglycine
GC: gastric cancer
IRF-1: interferon regulatory factor-1
lncRNA: long noncoding RNA
OS: overall survival
ROC: receiver operating characteristic
YY1: Yin Yang 1
