# Supplementary material for: A DCS-related lncRNA signature predicts the prognosis and chemotherapeutic response of patients with gastric cancer
Source: Biosci Rep. 2022 Sep 2;42(9):BSR20220989. doi: 10.1042/BSR20220989 (PMC9446389; doi:10.1042/BSR20220989)
Supplement: Supplementary Figures S1-S3 and Supplementary Tables S1-S3 [file BSR-2022-0989_supp.pdf]

(a)

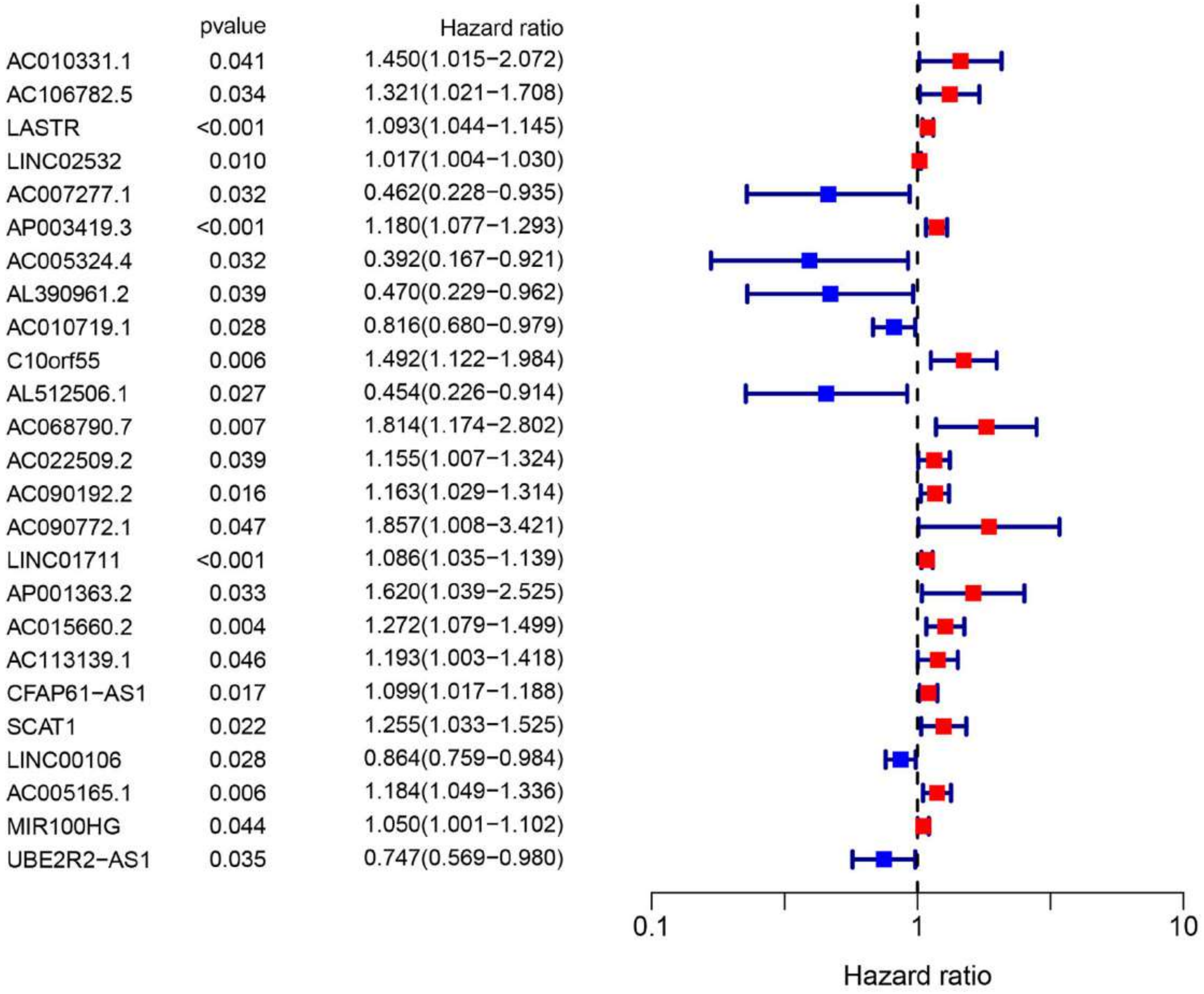
pvalue
Hazard ratio
AC010331.1 0.041 1.450(1.015−2.072)
AC106782.5 0.034 1.321(1.021−1.708)
LASTR <0.001 1.093(1.044−1.145)
LINC02532 0.010 1.017(1.004−1.030)
AC007277.1 0.032 0.462(0.228−0.935)
AP003419.3 <0.001 1.180(1.077−1.293)
AC005324.4 0.032 0.392(0.167−0.921)
AL390961.2 0.039 0.470(0.229−0.962)
AC010719.1 0.028 0.816(0.680−0.979)
C10orf55 0.006 1.492(1.122−1.984)
AL512506.1 0.027 0.454(0.226−0.914)
AC068790.7 0.007 1.814(1.174−2.802)
AC022509.2 0.039 1.155(1.007−1.324)
AC090192.2 0.016 1.163(1.029−1.314)
AC090772.1 0.047 1.857(1.008−3.421)
LINC01711 <0.001 1.086(1.035−1.139)
AP001363.2 0.033 1.620(1.039−2.525)
AC015660.2 0.004 1.272(1.079−1.499)
AC113139.1 0.046 1.193(1.003−1.418)
CFAP61−AS1 0.017 1.099(1.017−1.188)
SCAT1 0.022 1.255(1.033−1.525)
LINC00106 0.028 0.864(0.759−0.984)
AC005165.1 0.006 1.184(1.049−1.336)
MIR100HG 0.044 1.050(1.001−1.102)
UBE2R2−AS1 0.035 0.747(0.569−0.980)
0.1
1
10
Hazard ratio

(b)

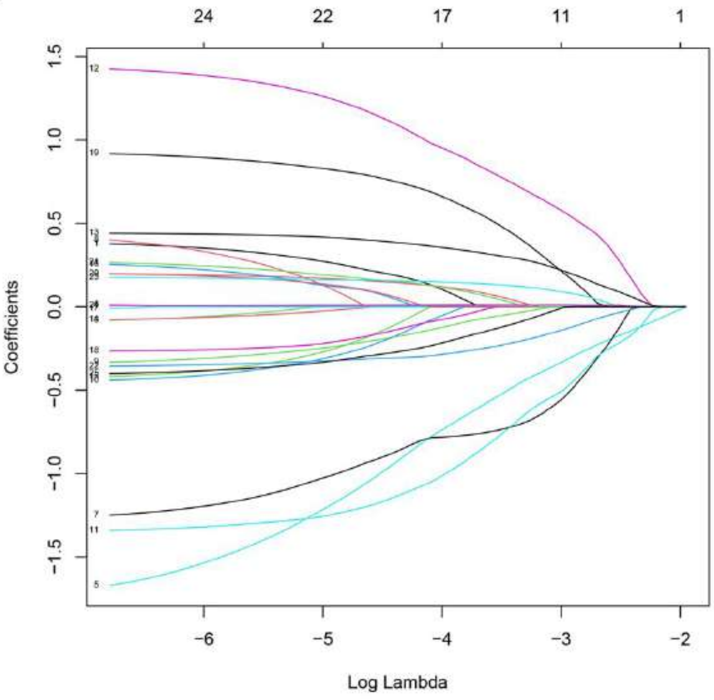
24
22
17
11
1
1.5
1.0
0.5
0.0
−0.5
−1.0
−1.5
Coefficients
−6
−5
−4
−3
−2
Log Lambda

(c)

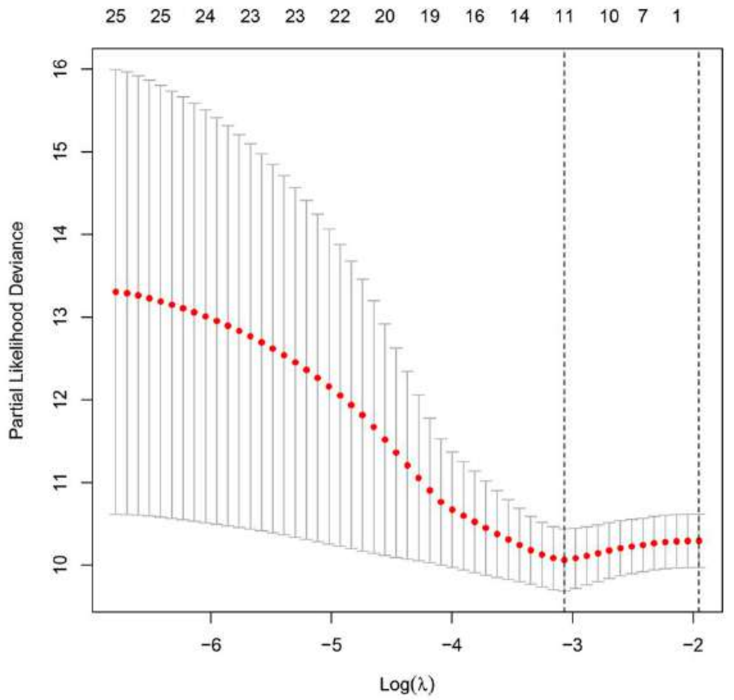
25 25 24 23 23 22 20 19 16 14 11 10 7 1
16
15
14
13
12
11
10
Partial Likelihood Deviance
−6
−5
−4
−3
−2
Log(λ)

(a)

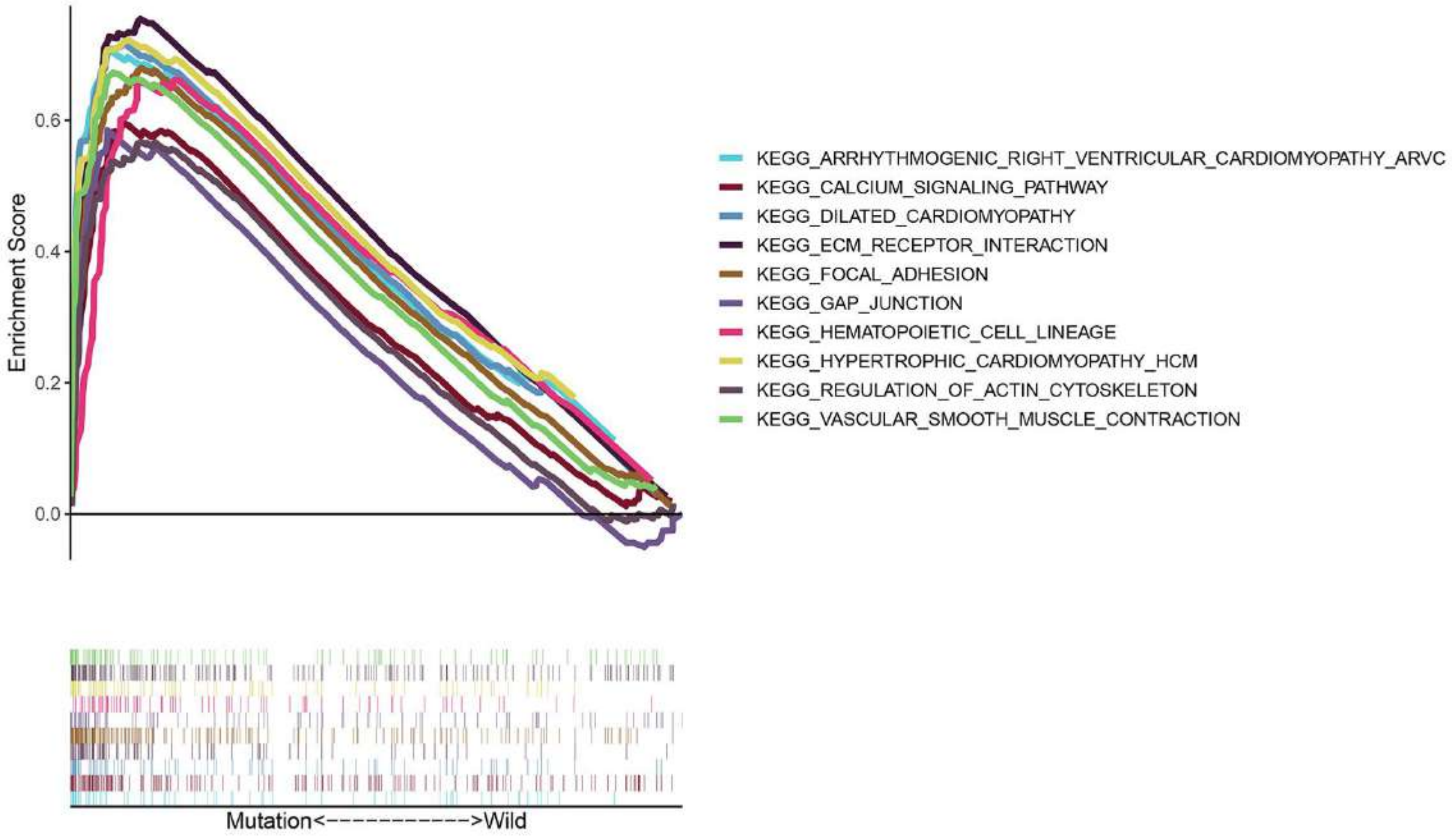
Enrichment Score
0.6
0.4
0.2
0.0
KEGG_ARRHYTHMOGENIC_RIGHT_VENTRICULAR_CARDIOMYOPATHY_ARVC
KEGG_CALCIUM_SIGNALING_PATHWAY
KEGG_DILATED_CARDIOMYOPATHY
KEGG_ECM_RECEPTOR_INTERACTION
KEGG_FOCAL_ADHESION
KEGG_GAP_JUNCTION
KEGG_HEMATOPOIETIC_CELL_LINEAGE
KEGG_HYPERTROPHIC_CARDIOMYOPATHY_HCM
KEGG_REGULATION_OF_ACTIN_CYTOSKELETON
KEGG_VASCULAR_SMOOTH_MUSCLE_CONTRACTION
Mutation<------------>Wild

(b)

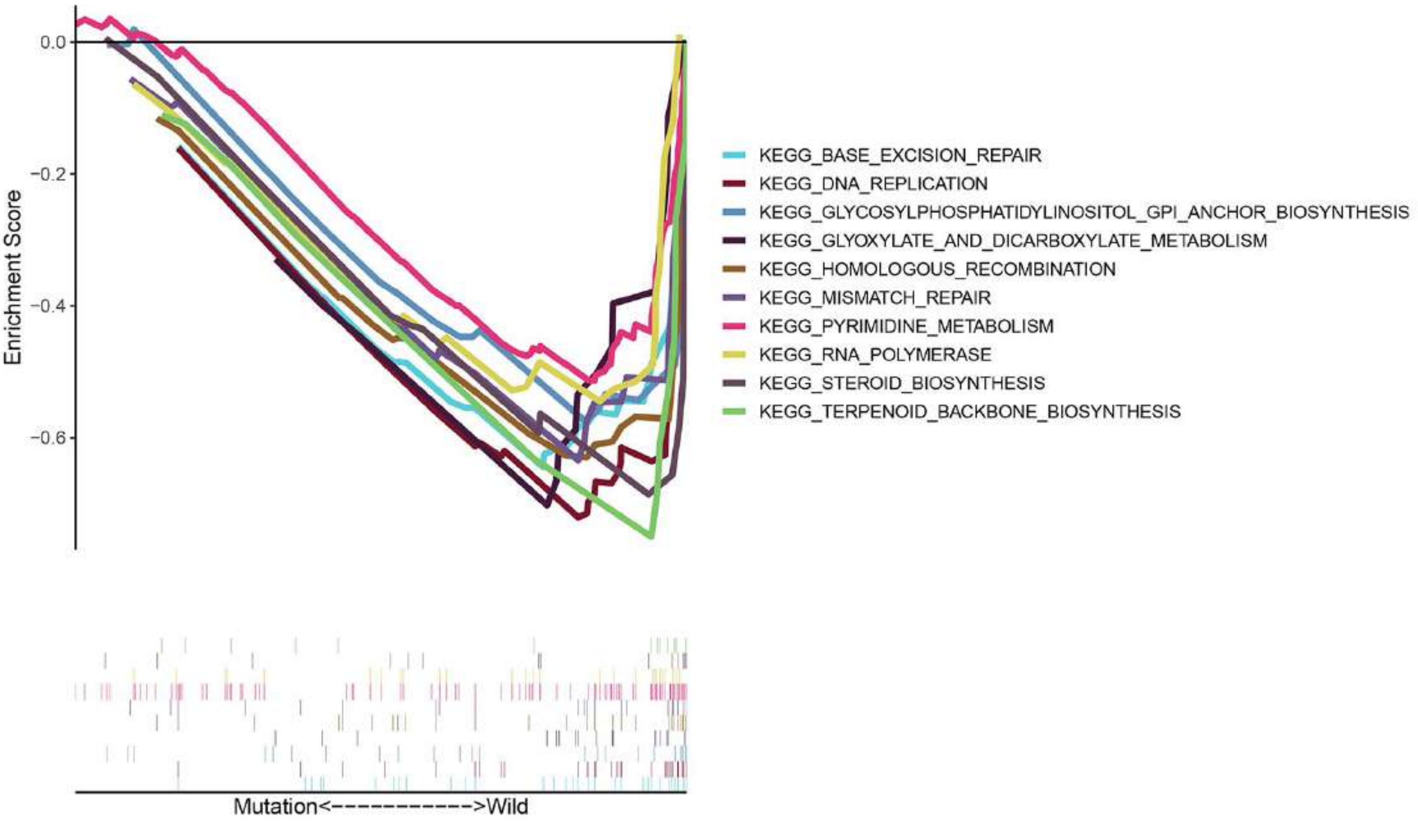
Enrichment Score
0.0
−0.2
−0.4
−0.6
KEGG_BASE_EXCISION_REPAIR
KEGG_DNA_REPLICATION
KEGG_GLYCOSYLPHOSPHATIDYLINOSITOL_GPI_ANCHOR_BIOSYNTHESIS
KEGG_GLYOXYLATE_AND_DICARBOXYLATE_METABOLISM
KEGG_HOMOLOGOUS_RECOMBINATION
KEGG_MISMATCH_REPAIR
KEGG_PYRIMIDINE_METABOLISM
KEGG_RNA_POLYMERASE
KEGG_STEROID_BIOSYNTHESIS
KEGG_TERPENOID_BACKBONE_BIOSYNTHESIS
Mutation<------------>Wild

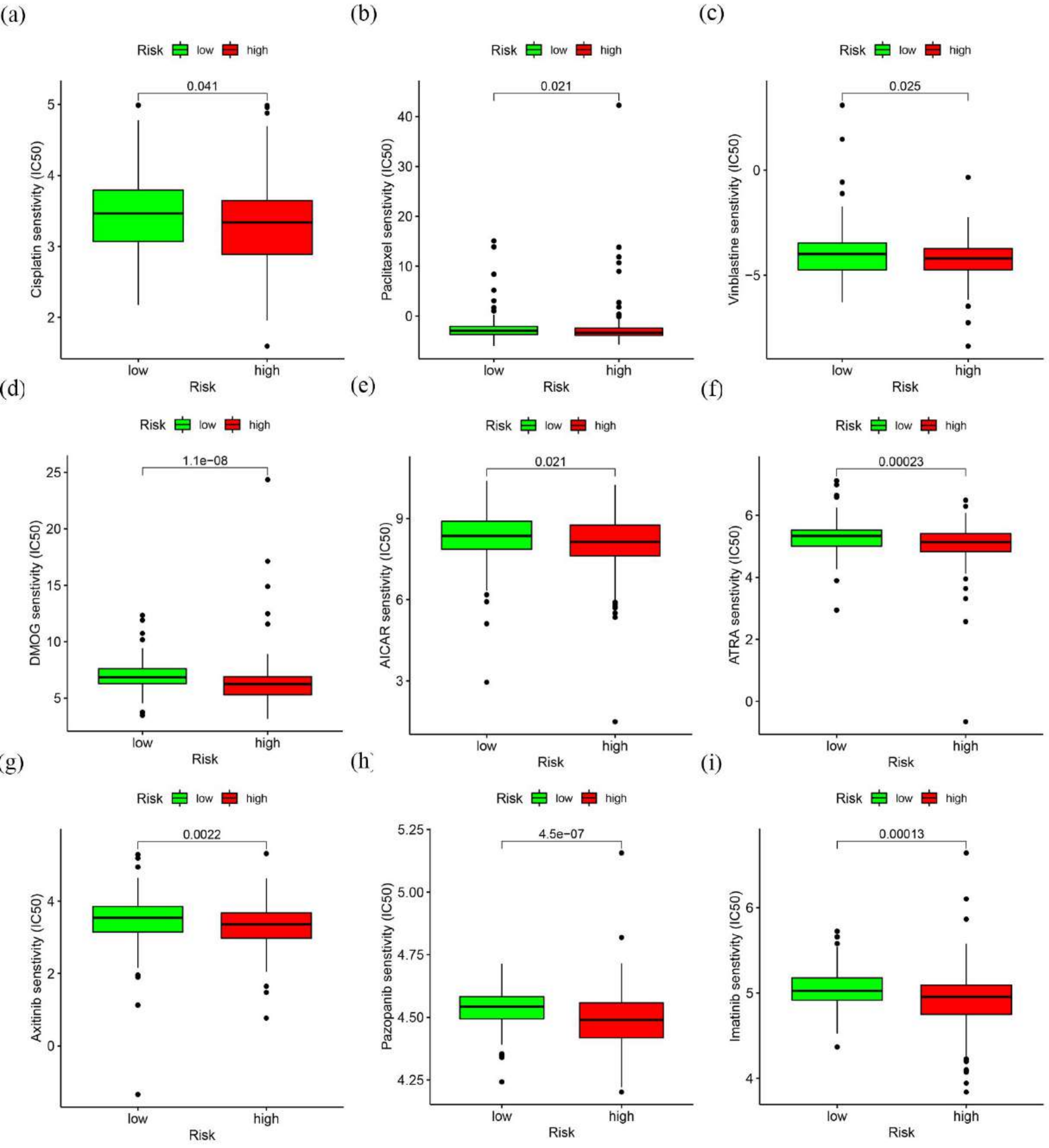

(a)
Risk low high
0.041
Cisplatin sensitivity (IC50)
low high
Risk
(b)
Risk low high
0.021
Paclitaxel sensitivity (IC50)
low high
Risk
(c)
Risk low high
0.025
Vinblastine sensitivity (IC50)
low high
Risk
(d)
Risk low high
1.1e−08
DMOG sensitivity (IC50)
low high
Risk
(e)
Risk low high
0.021
AICAR sensitivity (IC50)
low high
Risk
(f)
Risk low high
0.00023
ATRA sensitivity (IC50)
low high
Risk
(g)
Risk low high
0.0022
Axitinib sensitivity (IC50)
low high
Risk
(h)
Risk low high
4.5e−07
Pazopanib sensitivity (IC50)
low high
Risk
(i)
Risk low high
0.00013
Imatinib sensitivity (IC50)
low high
Risk

Table S1. 16 DCS therapy-related mRNAs obtained from the TCGA differential mRNA expression data

mRNA	LogFC	P-value	FDR
ACACB	-1.3248688	7.32094E-08	3.34018E-07
ITGB8	1.445876977	6.29695E-09	3.53598E-08
PTPRU	2.00668175	1.05044E-07	4.51069E-07
SH3BGR	-2.061212597	6.67707E-06	1.74081E-05
SRCIN1	2.231121404	2.62758E-06	7.37743E-06
PRKX	1.089757554	3.08548E-09	1.877E-08
NSUN5P2	1.083789752	1.74922E-07	6.08062E-07
F2	4.157425263	0.003763628	0.005972714
SNRPN	-1.298898296	9.70659E-10	7.87312E-09
STIP1	1.014221568	5.56244E-14	2.03029E-12
FKBP5	-1.158139919	0.001935847	0.003446751
PAIP2B	-1.25128054	9.28587E-10	7.87312E-09
MST1P2	1.231101821	0.003090037	0.005012726
ARPC1B	1.09404297	1.62051E-07	5.91485E-07
CCSAP	1.273562179	1.62966E-14	1.18965E-12

C2	1.717346756	1.78037E-08	8.66445E-08

Docetaxel, Cisplatin, and S-1 (DCS); Logarithmic fold change (log FC); False Discover Rate (FDR); The Cancer Genome Atlas (TCGA)

Table S2. Univariate Cox regression analysis of the 25 lncRNAs.

LncRNA	HR	HR. 95L	HR. 95H	P-value
AC010331.1	1.450264253	1.015129274	2.07191976	0.041105376
AC106782.5	1.320581056	1.021103779	1.707891365	0.034080565
LASTR	1.092996637	1.043621084	1.144708234	0.000163083
LINC02532	1.017069597	1.00399293	1.030316583	0.010361537
AC007277.1	0.462045024	0.228441035	0.934532645	0.031684776
AP003419.3	1.180109977	1.077287606	1.292746292	0.000370065
AC005324.4	0.392454387	0.16730497	0.920596955	0.031543293
AL390961.2	0.469620845	0.229285919	0.961872141	0.038806359
AC010719.1	0.815983895	0.68032173	0.978698296	0.028375202
C10orf55	1.492415263	1.122446426	1.984329287	0.005875345
AL512506.1	0.454226584	0.225732822	0.914008819	0.026967101
AC068790.7	1.813849892	1.174215903	2.801913537	0.007278533
AC022509.2	1.154946217	1.007208139	1.324354632	0.039130454
AC090192.2	1.162832241	1.028840854	1.314274035	0.015728482
AC090772.1	1.856887946	1.007776843	3.421424958	0.04716587

LINC01711	1.085760307	1.035078567	1.138923634	0.000742029
AP001363.2	1.619920064	1.039355835	2.524776333	0.033134613
AC015660.2	1.271853835	1.079410149	1.498607531	0.00406777
AC113139.1	1.192778103	1.003008509	1.418452176	0.046158413
CFAP61-AS1	1.099270712	1.016843122	1.188380068	0.017314058
SCAT1	1.254774403	1.032610232	1.524736783	0.02244666
LINC00106	0.864099067	0.758611674	0.984254821	0.027886857
AC005165.1	1.183779794	1.049009719	1.335864267	0.00622203
MIR100HG	1.050374057	1.001351308	1.101796793	0.043869086
UBE2R2-AS1	0.74657233	0.568628725	0.980200649	0.035385049

Hazard Ratio (HR); Long non-coding RNAs (lncRNAs)

Table S3. Top ten pathways enriched in the high-risk group and low-risk group.

GS<br>follow link to MSigDB	SIZE	NES	NOM p-vale	FDR q-vale
KEGG_HYPERTROPHIC_CARDIOMYOPATHY_HCM	83	2.49	0.000	0.000
KEGG_DILATED_CARDIOMYOPATHY	90	2.47	0.000	0.000
KEGG_VASCULAR_SMOOTH_MUSCLE_CONTRACTION	115	2.35	0.000	0.000
KEGG_ARRHYTHMOGENIC_RIGHT_VENTRICULAR_CARDIOMYOPATHY_ARVC	74	2.33	0.000	0.001
KEGG_CALCIUM_SIGNALING_PATHWAY	178	2.32	0.000	0.000
KEGG_FOCAL_ADHESION	199	2.27	0.000	0.001
KEGG_ECM_RECEPTOR_INTERACTION	84	2.25	0.000	0.001
KEGG_REGULATION_OF_ACTIN_CYTOSKELETON	213	2.16	0.000	0.003
KEGG_GAP_JUNCTION	90	2.12	0.000	0.004
KEGG_HEMATOPOIETIC_CELL_LINEAGE	85	2.05	0.004	0.008
KEGG_TERPENOID_BACKBONE_BIOSYNTHESIS	15	-1.97	0.002	0.130
KEGG_GLYOXYLATE_AND_DICARBOXYLATE_METABOLISM	16	-1.86	0.012	0.179
KEGG_BASE_EXCISION_REPAIR	35	-1.82	0.016	0.159
KEGG_STEROID_BIOSYNTHESIS	17	-1.80	0.010	0.146

KEGG_DNA_REPLICATION	36	-1.74	0.032	0.180
KEGG_HOMOLOGOUS_RECOMBINATION	28	-1.73	0.012	0.166
KEGG_PYRIMIDINE_METABOLISM	97	-1.69	0.047	0.179
KEGG_GLYCOSYLPHOSPHATIDYLINOSITOL_GPI_ANCHOR_BIOSYNTHESIS	25	-1.67	0.037	0.185
KEGG_MISMATCH_REPAIR	23	-1.60	0.064	0.248
KEGG_RNA_POLYMERASE	28	-1.60	0.066	0.224

NES: normalized enrichment score; NOM: nominal; FDR: false discovery rate. Gene sets with NOM p-value < 0.05 and FDR q-value < 0.05 are considered as significant.
